# Genomics-Driven Immunotherapy: Advancing Cancer Treatment through Personalized Approaches

**DOI:** 10.2174/0113892029403241260416110923

**Published:** 2026-04-17

**Authors:** Aditi A. Kate, Sharav A. Desai, Siddhi P. Sapkal, Madhulika M. More, Vipul P. Patel

**Affiliations:** 1 Department of Pharmaceutical Biotechnology, Sanjivani College of Pharmaceutical Education & Research, Savitribai Phule Pune University, Kopargaon, Maharashtra, India

**Keywords:** Tumor, tumor microenvironment, cancer immunotherapy, genomics, genomic biomarkers, precision medicine

## Abstract

Cancer is a complex disease involving the abnormal growth and dissemination of cells. The traditional treatments of cancer suffer from challenges like drug resistance and non-specificity. However, immunotherapy has become a promising alternative, leveraging the body's immune system to attack cancer cells. However, the variability in patients' responses to immunotherapy underscores the significance of personalized treatment strategies. Genomics is key to understanding how genetic changes drive tumor development and response to immunotherapy. Genomic profiling techniques, such as NGS, enable the identification of molecular biomarkers, such as Tumor Mutational Burden (TMB) and Microsatellite Instability (MSI), that predict response to immunotherapy. Furthermore, the Tumor Microenvironment (TME), consisting of different cells and molecules surrounding the tumor, plays an important role in cancer progression and treatment outcomes. In this review, we highlight how genomics-driven approaches are accelerating cancer immunotherapy. It presents how genomics can be used to personalize immunotherapy for cancer and discusses challenges such as tumor heterogeneity and resistance to therapy, which require innovative technologies such as single-cell RNA sequencing, AI, and machine learning in immunogenomics, and liquid biopsies to refine treatment strategies. In the future, the incorporation of CRISPR-based gene editing, personalized neoantigen vaccines, and combination therapies holds great promise for transforming cancer treatment.

## 1 INTRODUCTION

Cancer is a complex and diverse disease characterized by the growth and spread of abnormal cells in the body. Currently, cancer has been one of the leading causes of death in many countries across the world. According to data published by the World Health Organization in 2020, cancer ranks as the second leading cause of death, accounting for over 10 million deaths. Nowadays, various factors, including population growth, aging population, changes in diet and lifestyles, and environmental factors, have contributed to increasing the incidence of cancer [1]. These cancer cell clusters can form either benign or malignant tumors. Benign tumors are slow-growing, non-malignant, and do not invade other tissues or organs of the body, but may cause problems if they become large or develop in sensitive areas. Malignant tumors, however, are cancerous; they can also extend to affect adjacent tissues and spread to other parts of the body through a process called metastasis. Such tumors are aggressive, invade tissues and organs, and usually demand emergency treatments [2].

The treatment of cancer is performed through several methods, namely radical surgery, radiotherapy, chemotherapy, targeted therapy, immunotherapy, and hormonal therapy. Sometimes, a combination therapy is used; for example, a patient undergoes surgery to remove the tumor and then undergoes radiation or chemotherapy to ensure the complete removal of the malignant cells [3]. However, cancer treatment has remained a complex process through which several issues emerge, the major one being drug resistance. Tumors that are often attacked by chemotherapy drugs also become resistant to the drugs, which makes them less effective in fighting cancer.

The chemotherapeutic agents selected are non-selective in their action, which leads to their toxicity and side effects. These drugs target rapidly dividing tissues characteristic of cancer, but they also affect healthy tissues that divide rapidly, including bone marrow, the gastrointestinal system, hair, and reproductive organs. Therefore, patients suffer from side effects such as myelosuppression (a reduction of blood cell production), gastrointestinal disturbances (nausea, vomiting, and diarrhea), alopecia (hair loss), and cases of infertility. In addition, chemotherapy drugs induce oxidative stress and generate free radicals that damage cellular components such as DNA, proteins, and lipids. This damage can lead to long-term effects, including secondary malignancies, nephrotoxicity, cardiotoxicity, and neurotoxicity [4]^1^.

Immunotherapy in cancer has revolutionized oncology practice by leveraging the immune system's ability to identify cancer cells. Approaches such as immune checkpoint inhibitors, adoptive cell transfer, and cancer vaccines have demonstrated high efficacy in treating diverse cancers [5]. However, treatment outcomes vary significantly, with some patients experiencing substantial improvements while others showing minimal benefit, posing a significant challenge in clinical oncology. This variability underscores the need for personalized treatment approaches tailored to the genetic and molecular characteristics of each tumor. Immunotherapy has introduced new hope in treating incurable cancers; however, the variability in patients’ responses to these therapies underscores their complexity. This disparity underscores the ongoing need for research and the refinement of personalized treatment plans to enhance efficacy and tailor approaches to each patient's unique genetic and molecular profile [6, 7].

Cancer genomics has proved to be a rich source of understanding how genetic changes affect the tumor and the immune system regarding cancer identification and treatment. New technologies such as WGS and NGS have identified genetic changes, neoantigen profiles, and TMBs that can predict a patient's response to immunotherapy. For example, a high TMB in tumors often generates more neoantigens, enabling better immune system recognition and treatment with immune checkpoint agents. On the other hand, tumors can be characterized as low TMB, which would not respond to immunotherapeutic agents and would require alternative or adjuvant treatment plans [8].

Furthermore, the tumor microenvironment, that is, the surrounding tissue of the tumor, influences immunotherapy treatment outcomes. Genomic and transcriptomic analysis of the tumor microenvironment, especially of tumor-associated macrophages and cancer-associated fibroblasts, indicates that genetic and epigenetic alterations can promote or hinder immune infiltration and activity. Some of these have given way to new approaches to treatment that entail altering the TME to favor a better immune response [9].

The relationship between genomic and tumor responses to immunotherapy will be explored in this review, and how genetic changes affect immune recognition, response, and resistance to immunotherapy will be examined. New concepts in the genomic field will also be discussed, and their potential to enhance selection criteria, treatment modalities in immunotherapy, and overall survival outcomes for cancer patients will be considered.

## 2 TUMOR MICROENVIRONMENT (TME)

### 2.1 Overview of the Tumor Microenvironment (TME)

The TME is a surrounding matrix that incorporates multiple structures interacting with the tumor cells and is involved in tumor development, progression, and reaction to treatment. TME comprises a diverse array of cellular and noncellular elements that comprise the immediate microenvironment within the cancer tissue and affect the behaviour of cancer cells and the pathophysiology of the cancer. The main constituents of TME include cancer-associated fibroblasts (CAFs), immune cells (T cells, macrophages, and dendritic cells), endothelial cells, and pericytes. The non-cellular elements are the Extracellular Matrix (ECM), signalling molecules, cytokines, and growth factors. These components, when combined, form a supportive niche that not only promotes tumor survival and invasion but also regulates immune responses in Favor of tumor entities. Knowledge of the features of the TME is crucial, as it has a direct bearing on the planning of therapies such as immunotherapy—the attempt to recapture the TME for anti-tumor immunity [10].

#### 2.1.1 Stromal Cells

Stromal cells have come to be known as multifaceted cells of the TME that interact with cancer cells, affecting their growth, progression, and response to treatment. In the TME, somatic cells, including CAFs, adipocytes, and MSCs, contribute to the formation of a tumor-promoting microenvironment. They release growth factors, cytokines, and extracellular matrix molecules that regulate cancer cell growth, invasion, and metastasis. Further, stromal cells, which shape the tissue microenvironment, can promote or inhibit immune responses, thereby influencing how the tumor responds to treatment. Hence, it is considered a good candidate for treatment due to its inhibitory effects on tumor-promoting functions [11, 12].

##### 2.1.1.1 Endothelial Cells

These cells constitute the endothelial lining of blood vessels and hence play a central role in angiogenesis. However, another function of ECS is to promote migration, invasion, and metastasis of cancer cells as well as angiogenesis. ECs are highly susceptible and can change their differentiation type [13]. In tumor progression, normal endothelial cells undergo phenotypic changes from endothelial to mesenchymal cells and subsequently to cancer-associated fibroblasts (CAFs). This transition from endothelial cells (ECs) to CAFs is mediated by TGF-β and BMP signaling and is associated with the breakdown of cell junctions, increased cell size, altered motility, and aberrant endothelial phenotypes. These processes play a vital role in mediating tumor cell movement and invasion of neoplastic cells.

Metastasis refers to the transfer of cancer cells from the primary tumor microenvironment (TME) to other parts of the body. Intravasation is the first phase, in which tumor cells detach from the primary tumor and enter the bloodstream. During intravasation, tumor cells interact with the endothelium, and this interaction alters the endothelial barrier, permitting tumor cell passage through gaps between endothelial cells [14, 15].

##### 2.1.1.2 Cancer-Associated Fibroblasts (CAFs)

CAFs are the most abundant stromal cell type in tumors. According to the findings, cancer-associated fibroblasts are the primary source of collagen for cells, influencing the surrounding tumor tissue, immune cells, and endothelial cells 16]. They have been reported to be associated with therapy resistance and higher rates of recurrence in many neoplastic diseases, hence worse outcomes. They release several cytokines and growth factors, including TGF-β, IL-6, exosomes, CXCL2, CCL7, HGF, IGF, and CTGF. These findings are consistent with the assertion that CAFs can contribute to tumor growth in an indirect manner through the induction of inflammation, production of pro-angiogenic molecules, release of growth factors, and remodeling of the extracellular matrix [17].

##### 2.1.1.3 Mesenchymal Stem Cells (MSCs)

MSCs have the ability to differentiate into numerous specializations, such as fibroblast-like cells and fat cells. In TME, MSCs can enhance tumor growth by releasing pro-survival cytokines and growth factors into the tumor microenvironment [18].

##### 2.1.1.4 Adipocytes

Cancer-associated adipocytes release lipids and adipokines, which affect cancer cell function and metabolism. These cells help regulate tumor metabolism and are involved in inflammation and tumor promotion [19].

##### 2.1.1.5 Pericytes

Pericytes are cells that surround endothelial cells within blood vessels, especially those related to small vessels like capillaries. These glycoproteins play important roles in supporting vessel structure, developmental progression, and function. They help maintain the structural integrity of blood vessels [20]. They interact with endothelial cells by releasing various factors that inhibit pathological neo-angiogenesis and reduce vascular permeability. Additionally, pericytes are actively involved in the angiogenic process due to their dual roles in both promoting and inhibiting angiogenesis. They release molecules that modulate the behaviour of endothelial cells and regulate the release of VEGF, a factor that promotes new blood vessel formation. In addition, pericytes contribute to immune regulation in tumors by interacting with immune cells. They can either enhance T cell infiltration or suppress immune cell activation, thereby influencing the body's ability to fight cancer cells [21].

#### 2.1.2 Immune Cells

Immune cells are an essential component of the Tumor Microenvironment (TME). There is a link between TMEs and immune cells: immune cells can both stimulate tumor progression and inhibit it. Many cancer types, such as colorectal, hepatocellular, and cervical cancer, share a common mechanism of tumor formation involving chronic inflammation induced by persistent infection. The interaction between the cellular and structural components of the TME facilitates the aggressive and metastatic spread of tumor cells to distant sites.

There is mounting evidence that, within the TME, innate immune cells (such as macrophages, neutrophils, Dendritic Cells (DCs), innate lymphocytes, myeloid-derived suppressor cells, and Natural Killer (NK) cells) and adaptive immune cells (such as T cells and B cells) are present and can aid in detecting and eliminating cancer cells by recognizing tumor-specific antigens on their surfaces. Specifically, Cytotoxic T Lymphocytes (CTLs), also known as CD8+ T cells, are considered a highly effective defense mechanism for tumor prevention and elimination [22]. The comparison of anti-tumor *versus* immunosuppressive tumor microenvironments is shown in Fig. (**1**).

##### 2.1.2.1 T Cells

Various T-cell populations within the TME influence tumorigenesis, each generating a unique T-cell receptor (TCR) to recognize specific antigens. Cytotoxic T-lymphocytes (CD8+ T cells) recognize tumor antigens on malignant cells and subsequently eliminate these neoplastic cells. A favorable cancer prognosis strongly correlates with elevated Th1 cell numbers in the TME. These pro-inflammatory cells aid CD8+ T cells by releasing essential cytokines, such as interleukin-2 (IL-2) and interferon-gamma (IFN-γ). CD4+ T cells can differentiate into various subtypes within the TME and manage a wide range of immune responses. Regulatory T cells (Tregs) reduce inflammatory reactions and control autoimmune processes to maintain immune homeostasis, and play a crucial role in supporting cancer cells through indirect interactions with stromal cells, including fibroblasts and endothelial cells, and the release of growth factors [23]. IL-2, primarily secreted by activated T cells, regulates the balance and activity of natural killer (NK) cells, while Tregs help regulate immune homeostasis. Tumor microenvironments can be categorized into three types based on their immune composition: immune-excluded, immune-infiltrated, and immune-silent. Immune-infiltrated tumors exhibit a stable distribution of immune cells within the TME, whereas immune-excluded tumors have T lymphocytes at the periphery without penetrating the TME. Immune-silent tumors lack immune cell infiltration, indicating a deficient immune response to malignancy [24, 25].

##### 2.1.2.2 B Cells

B lymphocytes, known as B-cells, are specialized subsets among other immune cells in the human body. They are required in antibody formation, antigen presentation, and cytokine secretion. These cells are primarily located at the peripheral edges of the tumors and are present in lymph nodes adjacent to the TME. While B-cell infiltration is usually lower than that of T-cells, emerging evidence indicates that B-cell function and presence are paramount for cancer development. Tumor-invading 'B cell structures are correlated with the favorable prognosis of ovarian, breast, and melanoma cancers and facilitate close relations between T and B cells. Cells also play an important role in the formation of tertiary lymphoid structures, or ectopic lymphoid tissues, that develop within the TME. These structures are associated with a favorable prognosis in ovarian, breast, and melanoma cancers, as they facilitate close interactions between T and B cells. B cell–mediated anti-tumor immunity involves antigen presentation to T cells and the secretion of cytokines, such as IFN-γ, which activate cytotoxic immune responses. On the other hand, B cells may also exhibit pro-tumor effects, and their presence in the TME has been associated with poor outcomes in prostate, bladder, and renal cell carcinomas. Regulatory B cells promote tumor progression by producing cytokines such as IL-10 and transforming growth factor beta (TGF-β), which enhance immunosuppressive characteristics in neutrophils, macrophages, and cytotoxic T cells [26-28].

##### 2.1.2.3 Tumor-Associated Macrophages (TAMs)

Macrophages are integral components of the innate immune system, playing essential roles in wound healing, tissue repair, immune response regulation, and pathogen elimination. These cells can be categorized into inflammatory M1 macrophages, which are involved in phagocytosis as well as cell killing, and immune-suppressive M2 macrophages, which support tissue repair and wound healing. In the TME, the M2 phenotype is promoted by hypoxia and cytokine release, particularly interleukin-4 (IL-4), facilitating tumor growth and progression [29, 30]. TAMs promote tumor progression through angiogenesis and lymphangiogenesis (*via* VEGF-A/VEGF-C), EMT, ECM remodelling and invasion (*via* STAT3/STAT6 and cathepsins), and inhibition of T cell activation *via* immune checkpoints, although in some cases they recruit NK cells and inhibit metastasis [31]. Therapeutic strategies targeting macrophages in immunotherapy include TAM depletion (*e.g*., emactuzumab, IMC-CS4), blocking their recruitment signals (*e.g., CCR2/CCR5 and CCR5 inhibitors), enhancing phagocytosis through checkpoint inhibitors (PD-1/PD-L1, CD47, CD40, and trastuzumab), and disrupting macrophage function* (IDO inhibitors). Additionally, preclinical studies have reported strong anti-tumor effects of CAR-macrophage therapy [32, 33].

##### 2.1.2.4 Dendritic Cells

Dendritic Cells (DCs) are crucial antigen-presenting cells in the immune system. They recognize, capture, and present antigens to T cells in secondary lymphoid organs, such as lymph nodes, thereby linking the innate and adaptive immune responses and initiating pathogen-specific T-cell activity. However, the TME influences DC function by providing signals that can either activate an immune response against tumor cells or promote tolerance towards them [34, 35]. In the inflammatory condition, CMPs produce monocytic DCs (moDCs) *via* Nur77, and in the absence of inflammation, they produce common DC progenitors (CDPs) that give rise tolasmacytoid DCs (pDCs) and conventional DCs (cDCs) [36]. The conventional DCs are categorized into cDC1 (CD141, DEC205, Clec9A, XCR1) and cDC2 (CD1c, CD1a, CD103), whereas pDCs are characterized by CD123, CD303, CD304, and CD45RA. Functionally, cDC1 are essential for cross-presentation of tumor antigens on MHC-I and for activating CD8+ T cells, as well as for enhancing NK cell cytotoxicity *via* IL-12 and IL-15, whereas cDC2 are heterogeneous and also involved in cross-presentation and T cell activation [37]. Conversely, pDCs tend to induce tumor immune tolerance by expressing PD-L1 and granzyme B and by activating Tregs. In contrast, moDCs have been shown to cross-present tumor antigens and activate CTL proliferation in melanoma models [38]. In oncology, dendritic cell (DC)-based approaches focus on enhancing antitumor immunity through *in vivo* activation with agonists or VEGF inhibitors, expansion using cytokines such as Flt3L to increase cDC1 populations, and their application as vaccines. Notably, DC vaccines, including DCVax-L (glioblastoma, phase III trial) and neoantigen-loaded DC vaccines combined with immunomodulators, have demonstrated promising activity in enhancing responses to checkpoint blockade and extending patient survival [39, 40].

##### 2.1.2.5 Natural Killer Cells

Natural Killer (NK) cells are members of the innate lymphoid family, which develops out of the hematopoietic stem cells in the bone marrow. They are present in blood and tissues and have two major subsets: D56^bright^CD16^dim^ (immature, cytokine-producing) and *CD56*^dim^*CD16*^bright^ (mature, highly cytotoxic) [41]. They are regulated by a balance between NKR activation and *KIR* inhibition. NK cells can kill tumor cells in the TME through natural cytotoxicity and Antibody-Dependent Cellular Cytotoxicity (ADCC), *via* perforin/granzyme or FasL/TRAIL-dependent apoptosis [42]. They also produce cytokines as well as chemokines (*e.g*., CCL3, CCL4, CCL5, CXCL8), which can recruit immune cells to suppress tumor growth. However, NK subsets, including *CD39*^+ NK cells that are less cytotoxic, are linked with tumor progression and unfavorable prognosis. NK cell-based immunotherapeutic strategies include IL-2, IL-15, IL-18, IL-21, monoclonal antibodies, adoptive transfer, signaling pathways, and genetic engineering. Agents such as ALT-803 (IL-15 agonist) demonstrate benefits in metastatic lung cancer, and combination with PD-1 blockade and CTLA-4 inhibition (telemumab) prevents NK dysfunction and improves survival [43]. Adoptive NK cell therapy has shown success in pediatric brain tumors, and genetically engineered CAR-NK cells have demonstrated promising results in preclinical models of ovarian, breast, prostate, and colon cancers [44, 45].

#### 2.1.3 Extracellular Matrix

The extracellular matrix (ECM) is a complex network of proteins and other macromolecules that provides structural and biochemical support to surrounding cells and plays important roles within the TME [46]. It not only provides a physical scaffold for cells but also serves as a key factor in promoting tumor cell dissemination. The ECM is composed of collagen, elastin, fibronectin, laminin, and proteoglycans, forming a scaffold that maintains tissue integrity. In solid tumors, ECM components can account for up to 60% of the tumor mass. High collagen deposition, along with significant fibroblast infiltration, results in desmoplasia, which is strongly associated with poor patient prognosis. The ECM is not solely structural; it is also involved in signaling events that regulate cellular behavior, promoting tumorigenesis, metastasis, and chemoresistance [47].

Most cell types within the TME synthesize ECM, although the main contributors are cancer-associated fibroblasts (CAFs). The ECM stores cytokines and growth factors such as VEGF, FGF, PDGFB, and TGF-β, which are released through the action of proteolytic enzymes, including matrix metalloproteinases (MMPs). MMPs are involved in remodeling the surrounding matrix to facilitate cancer development and invasion. At advanced stages of cancer, more pronounced alterations occur in the ECM, and MMP-mediated degradation enables cancer cells to invade surrounding tissues, making the ECM a key factor in promoting tumor growth, metastasis, and resistance to treatment.

#### 2.1.4 Cytokines and Chemokines

These are signalling molecules secreted by cancer cells, stromal cells, and immune cells. These are molecules released by cells, such as cancer cells, stromal cells, or immune cells. They control the immune response, inflammation, and actions of dysfunctional cells present in the TME. For instance, TGF-β and *IL-6* are common immune suppressors and promoters of tumor growth [48, 49].

### 2.2 Impact of TME on Cancer Progression and Treatment Resistance

The Tumor Microenvironment (TME) is responsible for several hallmarks of cancer progression by providing a milieu conducive to cancer cell proliferation. The structural and functional integrity of the TME is maintained through interactions between cancer cells and surrounding stromal components, including Cancer-Associated Fibroblasts (CAFs), through the synthesis of growth factors and cytokines and the remodeling of the extracellular matrix (ECM).

Hypoxia within the tumor activates Hypoxia-Inducible Factors (HIFs) and VEGF, resulting in abnormal vascular patterns that promote cancer metastasis. Furthermore, immune cells within the TME can undergo phenotypic changes and acquire immunosuppressive functions; for example, Tumor-Associated Macrophages (TAMs) promote tumor proliferation and angiogenesis [50].

The TME significantly contributes to cancer treatment resistance by creating physical barriers to therapeutics, modulating immune responses, and initiating cellular processes that reduce the efficacy of both targeted and conventional treatments.

#### 2.2.1 Physical Barriers to Drug Penetration

The TME is densely populated with ECM components such as collagen and hyaluronan, as well as other large molecules. This arrangement provided a physical obstacle to drug access, especially to the innermost part of the tumor. Additionally, the poorly developed tumor vasculature results in irregular, immature, and leaky vessels, leading to uneven drug distribution within tumor cells and increasing cancer cell resistance to treatment [51].

#### 2.2.2 Immune Evasion Within the TME

The TME also plays a crucial role in immunosuppression to ensure the cancer cells are not attacked by the immune system. Tumor cells recruit regulatory T cells (Tregs) and Myeloid-Derived Suppressor Cells (MDSCs), which secrete inhibitory cytokines such as TGF-β, *IL-10,* and *IL-6.* Furthermore, tumor cells already bear immune checkpoints such as PD-L1, which constrain the actions of T cells and thus minimize immunotherapy impact. Additionally, lactate produced by cancer cells creates an acidic environment that inhibits T cell proliferation [52].

#### 2.2.3 Developing Resistance to Targeted and Conventional Therapies

The TME also promotes treatment resistance by elevating Hypoxia-Inducible Factors (HIFs), which enhance the activity of efflux pumps, leading to reduced intracellular drug concentrations under hypoxic conditions. CAFs create significant obstacles to therapy by secreting growth factors that activate survival signalling pathways and by synthesizing extracellular matrix components that trap drugs, thereby reducing their ability to reach tumor cells [53].

The tumor microenvironment is a complex and essential player in cancer development, progression, metastasis, and response to therapy. Understanding the interactions between cancer cells and their microenvironment opens the door to developing more effective, targeted therapies that not only attack cancer cells but also modify the TME to improve therapeutic outcomes. The TME's impact on treatment resistance is a key challenge in effective cancer therapy, as it provides multiple protective mechanisms for tumor cells against various therapeutic modalities. Addressing these factors is crucial for improving treatment outcomes in cancer patients.

Genomics and the Tumor Microenvironment (TME) are intricately linked in cancer progression and treatment, as genomic alterations in tumor cells significantly shape the TME, thereby influencing tumor growth, metastasis, and treatment responses. This relationship highlights the importance of integrating genetic analysis with TME studies to develop more effective therapeutic strategies, particularly for patients with resistant or metastatic cancers.

Since the TME is tightly regulated by genetic and epigenetic alterations, we next discuss how cancer genomics contributes to immunotherapy responses.

## 3 GENOMICS IN CANCER

Genomics^2^ refers to the study of the entire genome and associated data, including genome-wide analyses such as mapping genotypes and phenotypes relative to the complete genetic profile of an organism, as well as examining gene interactions and their effects with environmental factors.

This approach is particularly relevant for diseases with multifactorial pathogenesis driven by genetic, environmental, and lifestyle factors, such as cancer, diabetes, and cardiovascular diseases.

Genomic methods enable investigators to address these diverse determinants more broadly [54]. In oncology, the concept of molecular diagnostics has increasingly been approached from a genomic perspective, focusing on genetic alterations that drive tumorigenesis, proliferation, and treatment response. Specifically, genomic profiling can help identify unique mutations, amplifications, deletions, or translocations that are rare in cancer cells but facilitate oncogenesis. Such information enables precise medication management, in which treatments target specific genetic alterations in an individual's tumor, thereby enhancing therapeutic outcomes [55].

### 3.1 Genomic Alterations in Cancer

#### 3.1.1 Genetic Mutations

Genetic mutations are permanent changes in the DNA strands within gene sequences. In cancer, these changes lead to the uncontrollable division of cells within tissues, resulting in the formation of tumors. Mutations in cancer can be broadly classified into two categories: driver mutations and passenger mutations [56]. Driver mutations are essential to carcinogenesis and are the primary oncogenic alterations that provide a survival benefit to cancer cells, specifically in terms of growth parameters that enable rapid cycling and escape from growth controls. These mutations are found in certain growth factor receptor genes and cell cycle regulatory genes, commonly referred to as oncogenes and tumor suppressor genes, with notable examples including *KRAS, EGFR,* and *BRAF*. In contrast, passenger mutations are not implicated in cancer progression or disease initiation; rather, they arise in the genomic sequence as a consequence of the inherent genomic instability of cancer cells. Although passenger mutations do not confer advantages for selective growth, they can serve as biomarkers for tumor progression [57].

Driver mutations play a critical role in cancer progression, with examples including *EGFR, KRAS,* and *TP53*. *EGFR* (Epidermal Growth Factor Receptor) is often overexpressed in non-small cell lung cancer (NSCLC). As a result of mutations, this protein becomes constantly active, triggering cell division through pathways such as MAPK and PI3K; these effects can be inhibited by drugs like erlotinib and gefitinib [58]. *KRAS* mutations, found in pancreatic, colorectal, and lung cancers, result in continuous activation of the protein, promoting cell proliferation *via* the RAS/MAPK and PI3K/AKT signalling pathways, and are associated with poor prognosis and resistance to therapies [59]. Mutations in *TP53*, a tumor suppressor gene, are present in approximately 53% of cancer cases. *TP53* encodes a protein that regulates the cell cycle, controls DNA repair, and initiates apoptosis. Mutations in this gene lead to genomic instability and contribute to cancer development.

#### 3.1.2 Genomic Instability and Mutational Signatures

One of the hallmarks of cancer cells is genomic instability, which leads to a high mutation rate and chromosomal aberrations. This instability can arise from defects in normal DNA repair mechanisms, such as Mismatch Repair (MMR) or Homologous Recombination (HR). Mutational signatures represent specific genomic alterations resulting from particular mutational processes, such as exposure to carcinogens or defects in DNA repair. For instance, MSI, resulting from MMR defects, is found in colorectal and endometrial cancers and serves as a biomarker for the use of immune checkpoint inhibitors [60].

#### 3.1.3 Epigenetic Modifications and their Role in Tumorigenesis

Epigenomic^3^ changes are selective and reversible modifications to genes that do not involve alterations in the DNA sequence and are also implicated in cancer. Notable changes include DNA methylation (shown in Fig. **2**), where high levels of methylation at the promoter region of a tumor suppressor gene, such as MLH1, lead to its suppression. Post-translational modifications of histone proteins, such as acetylation or methylation, result in changes in chromatin conformation, which can either promote or inhibit gene expression. Chromatin remodeling is a process that modulates chromatin accessibility and influences transcription, and is associated with cancers such as lymphoma and leukemia. These epigenetic alterations typically enhance cancer development when combined with genetic changes [61].

### 3.2 Genomics of the Tumor Microenvironment

The TME comprises various cell types that support tumor growth, including CAFs, immune cells, endothelial cells, and stromal cells. Molecular characterization of these TME components has revealed that non-tumoural cells also undergo somatic genomic changes, leading to abnormal behaviour within the TME. For instance, CAFs may undergo genomic changes that trigger the release of growth factors and cytokines to promote tumor growth, metastasis, and resistance to therapy. Likewise, Tumor-Associated Macrophages (TAMs) exhibit epigenetic changes, rendering them less sensitive to immune surveillance by tumor cells [62].

These analyses also reveal how the genomics of tumor cells and TME elements influence oncological behaviours and treatment outcomes. Oncogenic cells can alter the TME in several ways, particularly when they harbor specific mutations, such as those in the *RAS* or *TP53* genes. These mutations secrete signalling factors. These mutations may produce pro-inflammatory cytokines and growth factors that mobilize and recondition CAFs and immune cells, thereby facilitating further tumorigenesis. This alters TME and produces facilitating signals, which allow tumor cells to survive, invade, and become resistant to treatments, thereby establishing a mutual relationship between tumor cells and the surrounding environment. Hence, there is a critical need to identify these genomic interactions in the TME so that novel approaches to cancer treatment can be designed and patients’ quality of life can be enhanced [63, 64].

### 3.3 Implications for Cancer Treatment

Molecular pathology offers a more precise approach to defining cancer at a molecular level and assists in the discovery of drugs for use in cancer treatment. It is therefore possible to identify driver mutations that characterize cancer and key signalling pathways in tumors in order to treat them accordingly. This has facilitated the process of determining an individual's treatment plan based on the specific details of their cancer. For example, PARP inhibitors such as Olaparib target cancer cells with BRCA1 or BRCA2 mutations and exploit defective DNA repair mechanisms to kill cancer cells. Tyrosine Kinase Inhibitors (TKIs), such as Erlotinib, are applied in NSCLC-affected persons with EGFR abnormalities, leading to a marginal increase in survival duration.

Targeted therapies have several benefits compared to conventional chemotherapy. According to researchers, they have fewer side effects since they only affect cancer cells and not the normal body cells. This has increased the focus on the specificity of treatment as well as the overall quality of the patient's life. The above-mentioned therapies may have an advantage over traditional chemotherapy by eradicating the primary molecular drivers of cancer. Therefore, genomic profiling and precision medicine can be considered highly effective approaches to more successful and personalized cancer treatment, opening new paths to improved patient outcomes [65].

These genomic alterations not only define tumor biology but also determine how patients respond to immunotherapy, thereby shaping current therapeutic strategies.

## 4 INTRODUCTION TO IMMUNOTHERAPY

Immunotherapy, which seeks to boost the body’s immune system to attack cancer cells, has been one of the most exciting advancements in cancer treatment. Modern basic and clinical investigation has demonstrated significant progress, despite the late nineteenth-century roots of the approach that harnesses the host immune system to combat cancer [66]. While several cancers have shown lasting clinical benefits from immunotherapy, most patients exhibit low overall response rates, and the mechanisms underlying successful immunotherapies remain poorly understood [67, 68]. Immune cells are core components of immunotherapy; as such, an understanding of immune infiltration within the TME is important for enhancing response rates and identifying new treatment approaches. Despite the tumor-immune ecosystem being characterized by a diverse range of cell types, advances in single-cell analysis techniques have made it possible to study the TME effectively [69]. Although substantial attention has been given to identifying T cells as effector cells associated with carcinogenesis and immunotherapy, other immune cells from both innate and adaptive immunity—including Dendritic Cells (DCs), macrophages, natural killer (NK) cells, and B cells—are also involved in tumor progression and response to immunotherapy. The goal of immunotherapy is to produce a therapeutic effect in diseases that are immune-mediated, such as immunodeficiencies, autoimmune diseases, organ and tissue transplants, infectious diseases, cancers, hypersensitivity reactions, and disorders of inflammation, and any other condition where immunotherapy may improve the quality of life [70].

### 4.1 Overview of Immunotherapy Approaches

The principal classifications of immunotherapy include oncolytic viruses, cancer vaccines, cytokines, adoptive T cell transfer, and immune checkpoint inhibitors. These various types of cancer immunotherapy have been developed and shown promise in clinical settings. The fundamental ideas behind each approach are clarified, along with their cellular and molecular bases at each stage. TCR (T-cell receptor), NK (natural killer) cells, CAR-T (chimeric antigen receptor T-cell), and DCs (dendritic cells) are all involved in these processes [6].

#### 4.1.1 Immune Checkpoint Inhibitors (ICI_s_)

Immune checkpoint inhibitors are immunotherapies that enhance the body's defences against tumors by targeting immune receptors located on the surface of T-lymphocytes [71]. Immunological checkpoints are cell surface receptors that regulate the activation or suppression of the immune response [72]. “T-cell exhaustion” refers to the condition in which effector T cells within the tumor exhibit reduced effector capacity and cytokine expression and become resistant to reactivation. These exhausted T cells extensively express inhibitory surface molecules such as LAG-3, PD-1, CTLA-4, and TIGIT, which effectively block T cell activation. These inhibitory surface molecules are commonly known as immunological checkpoints [73]. ICIs that target these co-inhibitory receptors, such as anti-PD-1, anti-CTLA-4, and anti-PD-L1, can bind to them and reactivate the immune system to target tumor cells, as shown in Fig. (**3**) [74, 75]. Some FDA-approved immune checkpoint inhibitors that are currently undergoing clinical studies are summarized in Table (**1**).

##### 4.1.1.1 CTLA-4 Inhibitor

CTLA-4, a molecule belonging to the immunoglobulin superfamily, is primarily associated with activated T cells and plays a key role in regulating the strength of T cell activation. It binds to the B7 receptor on the surface of B cells and Antigen-Presenting Cells (APCs), but unlike CD28, it downregulates T cells, thereby reducing antibody production and preventing allograft rejection. CTLA-4 expression peaks 2–3 days after T cell stimulation, whereas CD28 is constitutively expressed on naïve T cells [84]. The absence of CTLA-4 leads to unrestricted T cell proliferation. Inhibiting CTLA-4 can directly enhance immune responses against tumors and, indirectly, boost effector T cell activity while reducing Treg-mediated immunosuppression. The FOXP3 gene controls CTLA-4 expression in Treg cells, and blocking CTLA-4 in Treg cells diminishes their ability to suppress both autoimmune and antitumor immunity, although the exact mechanism remains unclear [85]. Overall, CTLA-4 inactivation is important as a target for antitumor immune modulation.

Yervoy (ipilimumab) is a monoclonal antibody of the IgG1 subtype that targets CTLA-4 to modulate immune responses. It was approved for melanoma treatment in 2011 and is also used in combination with nivolumab to treat advanced renal cell carcinoma, metastatic colorectal cancer (MSI-H/dMMR), malignant pleural mesothelioma, NSCLC, and hepatocellular carcinoma [86]. In 2020, the FDA approved nivolumab combined with ipilimumab for treating unresectable malignant pleural mesothelioma in adults and metastatic NSCLC with PD-L1 expression ≥1%, without EGFR/ALK gene mutations. However, since CTLA-4 plays a critical role in regulating immune responses, ipilimumab administration can cause immune-related adverse effects (irAEs), such as colitis and enterocolitis [87].

##### 4.1.1.2 PD-1 Inhibitor

The second immune checkpoint is PDCD1 or CD279, *i.e*., the PD-1 pathway. PD-1 regulates T cell-mediated response by playing an important role in the programmed cell death signalling process [88]. Two receptor ligands, namely PD-L1 and PD-L2, suppress anti-tumor immunity by binding to T cells and preventing T cell proliferation, activation, and cytotoxic release in cancer. Within the TME, immune tolerance is induced and maintained by the PD-1/PD-L1 pathway. Different types of immune cells, such as dendritic cells, activated monocytes, natural killer cells, B cells, and T cells inside the TME, have been shown to express PD-1 [89]. Various immunotherapies that increase the patient’s immune system response using the PD-1 pathway have been established and have changed the landscape of treatment in several types of cancer, such as NSCLC, melanoma, MCCs, and HNSCCs. The US FDA approved three monoclonal antibodies: Nivolumab, Pembrolizumab, and Cemiplimab for use in inhibiting PD-1 [90].

Nivolumab is the first mAb inhibitor, which is a fully human immunoglobulin G4 (IgG4) type and selectively neutralizes PD-1 through interaction with its ligands, PD-L1 and PD-L2. It has also been found that tumor cells evade immune system surveillance through regulation of the PD-1/PD-L1 signalling system, and as a result, cellular immunity is reduced [91]. Nivolumab did not show any significant adverse effect in patients with mRCC; however, it is associated with several immune-related adverse events, including renal, pulmonary, rheumatological, gastrointestinal, hepatic, dermatological, and endocrine reactions [92].

Pembrolizumab is another humanized IgG4 mAb targeting the PD-1/PD-L1 axis across various types of cancer. The FDA approved pembrolizumab for the treatment of several tumor types due to its excellent objective response rates, good pharmacokinetic profile, and safety record [93]. The FDA announced on October 13, 2021, that patients with recurrent metastatic cervical cancer whose tumors have PD-L1 expression of 1% or higher may benefit from pembrolizumab in combination with chemotherapy, with or without bevacizumab. While pembrolizumab is effective in targeting the tumor itself, it also modulates the immune system, eliciting strong responses across various tumor types, based on multiple studies. The first tissue- and site-agnostic medication, pembrolizumab, was recently approved by the FDA for the treatment of patients with dMMR/MSI-H tumors [94].

Cemiplimab is a humanized IgG4 mAb indicated for the treatment of patients with locally advanced or metastatic CSCC who are not suitable candidates for curative surgery or radiotherapy. It was approved by the European Medicines Agency (EMA) in June 2019 and by the FDA in September 2018, respectively [95]. Cemiplimab has shown marked anti-tumor efficacy with reasonable tolerability in patients with mCSCC. The most common adverse events reported in patients with CSCC receiving or undergoing treatment with cemiplimab (3 mg/kg) were fatigue (27.0%) and diarrhea (23.5%). Cemiplimab offers significant potential for treating CSCC patients, as evidenced by its association with better OS and PFS in CSCC patients compared with EGFR inhibitors and chemotherapy [96].

##### 4.1.1.3 PD-L1 Inhibitor

There are two types of PD-1 ligands: PD-L1 and PD-L2. Both tumor and immune cells can be PD-L1 positive, which serves as a biomarker to predict a patient’s response to antibodies targeting PD-1 in various cancer types [97]. PD-L1 interacts with T-cell negative co-stimulatory molecules such as PD-1 and B7, contributing to the inhibition of the cancer-immunity cycle [98]. This binding prevents T cell migration and proliferation, ultimately limiting the destruction of tumor cells. The PD-L1 inhibitors approved by the US FDA to date include atezolizumab, durvalumab, and avelumab, which have been used to treat several types of solid tumors, including NSCLC, melanoma, Merkel Cell Carcinoma (MCC), and Head and Neck Squamous Cell Carcinoma (HNSCC).

Atezolizumab is an IgG1 monoclonal antibody designed to reduce Fc-mediated antibody-dependent cellular toxicity, thereby preventing the elimination of T cells expressing PD-L1 [99]. It received US FDA approval in October 2021 as an adjuvant treatment for stage II and IIIA NSCLC in patients with PD-L1 expression of ≥1% on tumor cells [100].

Avelumab (MSB0010718C) is a fully human IgG1 monoclonal antibody targeting PD-L1, inhibiting its interaction with PD-1 [101]. Avelumab was subsequently approved by the FDA in March 2017 for use in patients with metastatic MCC, regardless of prior treatment [101]. In November 2015, the FDA designated avelumab as a breakthrough therapy for treating patients with metastatic MCC whose disease had progressed after chemotherapy, based on data from Part A of the JAVELIN Merkel 200 clinical trial [102].

Durvalumab (MEDI4736) is another immunotherapy approved by the FDA. It selectively targets PD-L1 with high affinity, preventing its binding to both *CD80* and PD-1 [103, 104]. The FDA recognized durvalumab as a breakthrough therapy in February 2016, based on phase I trial data. The trial targeted patients with metastatic urothelial bladder cancer whose tumor cells were PD-L1 positive and had progressed after receiving a standard platinum-containing regimen or during treatment [105].

#### 4.1.2 Adoptive Cell Therapy

Adoptive Cell Therapy (ACT) is an immunotherapy technique used to enhance the immune system's ability to combat tumor cells. T cells play a central role in this treatment due to their capacity to directly target and destroy cancerous tissues. In this approach, T cells are harvested either from a tumor sample or the blood of cancer patients. These cells are then modified in the laboratory to improve their ability to recognize cancer cells before being infused back into the patient’s body [106]. However, merely increasing the number of T cells may not always be sufficient to completely eradicate cancer. For effective treatment, killer T cells not only need to be present in large numbers at the tumor site but must also remain activated long enough to eliminate all tumor cells.

One form of ACT, called Tumor-Infiltrating Lymphocyte (TIL) therapy, involves collecting, expanding, and reinfusing T cells derived from cancer patients. Despite its potential, TIL therapy has limitations, including challenges with enrolling enough patients. To address this, peripheral lymphocytes are used to develop engineered T Cell Receptor (TCR) therapies that enhance the anti-tumor activity of T cells by enabling them to target specific cancer antigens. However, T cells typically attack cancer cells that express their specific antigens. To overcome this, chimeric antigen receptor (CAR) T cells can be used. CAR-T cells are genetically engineered to recognize and kill cancer cells, regardless of their antigen expression. This personalized medicine approach has been approved by both the FDA and EMA for the treatment of lymphoma [107].

Currently, the three primary ACT techniques are TILs, chimeric antigen receptor (CAR) T cells, and genetically modified T-cell receptors (TCRs). TIL therapy involves the expansion of a diverse population of autologous T cells extracted from a tumor, while CAR-T and TCR therapies focus on the propagation of genetically modified T cells designed to target specific antigens. Although hematologic malignancies have shown promising responses to ACT, further research and refinement are needed to improve its effectiveness across a broader range of cancers [108]. The table below (Table **2**) outlines the treatment modalities for adoptive cell therapy.

##### 4.1.2.1 CAR-T cell and TCR T Cell

In 2017, the FDA approved CAR-T cell therapy for treating B-acute lymphoblastic leukaemia (B-ALL) in patients under 25 years old. CAR-T cells are genetically modified T cells that express antigen-specific receptors, independent of MHC restriction. Known as a “living drug,” these modified T cells are stable, reproduce in the body, and can identify and destroy malignant cells if they recur, offering the potential for long-lasting results from a single treatment (Fig. **4**) [109].

CAR-T-cell therapies use genetically engineered T cells that recognize specific antigens on cancer cell surfaces through antibody fragments. Initially, the first-generation CAR-T cells involved modifying T cells to express immunoglobulin-TCR chimeric molecules, but these cells had limited persistence in the body. This issue was addressed by introducing costimulatory molecules such as *CD28* into CARs, enabling the modified T cells to survive longer and remain active. This innovation led to significant clinical results, including CD19-specific CAR-T cells with dual CD28/CD3-zeta signalling, which induced molecular remissions in adult patients with Acute Lymphoblastic Leukaemia (ALL) [110].

Other signalling molecules were also tested for efficacy, with Porter *et al.* showing that autologous T cells modified to target CD19, using CD3-zeta and 4-1BB signalling domains, could produce potent CD19-specific immune responses in patients with chronic lymphocytic leukaemia (CLL). These findings highlighted the promising potential of CAR-T therapies in treating blood cancers, with CD19-targeting CAR-T cells showing strong antitumor activity [111].

Meanwhile, TCR-T cell therapy involves the engineering of T cells to express specific T-Cell Receptors (TCRs) to target cancer antigens. It was demonstrated that transferring TCR genes into Peripheral Blood Lymphocytes (PBLs) from melanoma patients could create T cells with antitumor activity. Clinical trials later confirmed the efficacy of TCR-T therapy, with tumor regression observed in metastatic melanoma patients. Specifically, genetically modified TCR-T cells target the cancer-testis antigen NY-ESO-1, which is overexpressed in tumor cells, and produce long-term antigen-specific antitumor responses, resulting in tumor regression [112].

Consequently, CAR-T and TCR-T therapies have shown significant promise, delivering strong clinical efficacy in cancer treatment. These therapies have proven most effective in haematological malignancies, such as leukaemia, and ongoing research aims to improve outcomes in solid tumors [113].

#### 4.1.3 Cancer Vaccine

Active immunotherapy has been replaced by therapeutic cancer vaccines as one of the effective treatments for certain cancer types. These vaccines are designed to induce an anti-tumor immune response. On one hand, they stimulate the immune system to target tumors, while on the other, they minimize adverse effects, allowing for cancer regression or eradication [114]. Despite significant investment in the research and development of cancer vaccines, creating and successfully implementing highly effective vaccines in clinical settings has remained a challenging task. Nevertheless, the U.S. Food and Drug Administration (FDA) has approved two vaccines for cancer prevention: one against the human papillomavirus (HPV), which causes cervical cancer, and another against the hepatitis B virus (HBV), which leads to liver cancer.

Cancer immunotherapy encompasses a wide range of innovative, potentially life-saving treatments that stimulate the immune system to target and eliminate tumors [115]. Therapeutic cancer vaccines can be categorized into two main types: cell-based and antigen-based vaccines. Cell-based vaccines utilize cell transfer to induce a specific anti-tumor immune response. In contrast, antigen-based vaccines aim to boost the body's defence mechanisms by either presenting an existing antigen in a novel way or introducing a new antigen [116]. The following table (Table **3**) outlines the different types of therapeutic vaccines, along with their mechanisms of action for specific cancer types [117].

#### 4.1.4 Oncolytic Virus Therapy

With the goal of stabilizing and slowing tumor growth, oncolytic viruses (OVs) are biological agents capable of identifying, entering, and destroying a variety of cells within the tumor microenvironment. They are genetically programmed to detect specific targets and have an innate tropism for tumor cells. Clinical trials are being conducted on a number of OVs as potential cancer treatments. Additionally, OVs also have the potential to regulate the formation of an anticancer response by stimulating the immune system against cancer cells [118].

##### 4.1.4.1 Oncolytic Viruses and their Mechanism of Action

Oncolytic viruses can be delivered intratumorally, subcutaneously, intraperitoneally, or intrathecally. Tumor cell tropism and genetic targeting drive virus localization to tumor cells. The virus binds to tumor-associated markers and infects host cells *via* specific receptors.

The virus employs the cellular machinery to perform genome replication, leading to the production of viral proteins, suppression of cellular function, induction of oxidative stress conditions, and activation of autophagic pathways. They also cause antigen-presenting cells to form endosomal vesicles that fuse with lysosomal vesicles; as a result, the virus is degraded, and smaller viral particles within the cell are processed.

This induces the expression of both MHC II and viral proteins on the cell surface, creating an antigenic environment that activates both CD4+ and CD8+ T cells. Tumor cells are destroyed by viral action as well as by the host immune response and viral particles within the organism. Cell death leads to the release of tumor antigens, resulting in the formation of a new inflammatory response. The following Fig. (**5**) shows the mechanism of action of oncolytic viruses.

##### 4.1.4.2 Clinical Experience with Oncolytic Viruses

###### 4.1.4.2.1 Esophageal Cancer

Endoscopy was used to administer OBP-301, a type 5 adenovirus, to patients with esophageal cancer. Following initial treatment, there was a brief, self-limited lymphopenia as a side effect; the desired response rate was 91.7%. No malignant cells were found in the biopsy, despite the observation of local CR (83.3% in stage I and 60% in stages II and III) and PR [119].

###### 4.1.4.2.2 Colorectal Cancer

For Respectable Colorectal Cancer (CRC), an engineered adenovirus called Enadenotucirev was used. Eighty percent of patients with high local CD8 infiltration in the tumor showed signs of an immune response. The delivery paths were both reasonable and efficient. The combination of Enadenotucirev with various immunotherapy modalities is supported by this phase 1 investigation [120].

###### 4.1.4.2.3 Pancreatic Cancer

VSV is an oncolytic virus used in pancreatic cancer treatment, with VCV-ΔM51 showing sensitivity and tolerance in pancreatic ductal adenocarcinoma cells. VSV-*p53*OV inhibited PDAC cell multiplication. However, VSV is susceptible to mutation and lacks proofreading. It is crucial to maintain genetic stability in beneficial transgenic pieces. A study found that all viruses maintained their original red fluorescent protein and p53 sequences after 33 generations [121].

### 4.2 Key Advantages and Challenges in Immunotherapy

Surgery, radiotherapy, and chemotherapy have been identified over the years as the three main cancer treatment approaches. However, with the success of immunotherapy, either alone or in combination with other approaches, immunotherapy has been acknowledged as the fourth critical pillar of a comprehensive tumor treatment strategy. Immunotherapy is a different form of cancer treatment compared to conventional chemical therapies that seek to contain the progression of cancer cells; instead, the immune system of the body is employed in immunotherapy to identify and destroy cancer cells. The majority of cancer treatments, such as surgery, radiotherapy, and chemotherapy, have been effective in treating primary carcinoma. However, disease relapse remains common, as residual malignant cells or metastatic tumors are often detected after treatment. For this reason, immunotherapy is one of the complementary or alternative treatment options. However, challenges in developing and translating immunotherapies include genetic instability in cancer cells, such as heterogeneity, altered ploidy, and various mutations. Additionally, clonal mutations and intra- and inter-tumor heterogeneity further complicate the effectiveness of personalized therapy approaches [122].

To bridge immunotherapy and genomics, researchers have identified key biomarkers that predict patient response.

## 5 GENOMIC BIOMARKERS AND PREDICTIVE INDICATORS

Genomics plays an essential role in understanding tumors' response to immunotherapy by revealing patient-specific mutations, tumor heterogeneity, and resistance mechanisms, all of which inform personalized therapy strategies. Research has demonstrated that genes and pathways predicting patient responses to immune checkpoint blockade (ICB) can be precisely identified using whole-exome sequencing (WES). Proteogenomic methods also use proteomic and genomic data to identify protein therapeutic targets for personalized therapy in patients with melanoma, discover over-represented pathways, and define mutational landscapes. Additionally, tumor-derived genetic changes can impact the host immune system and the tumor microenvironment, thereby affecting the effectiveness of immunotherapy. This demonstrates the significance of identifying somatic mutations linked to tumor immunity in order to predict how patients will respond to immune checkpoint inhibitors [123].

Genomic markers are essential for predicting the response to immunotherapy in various types of cancer. Biomarkers are biological indicators used to identify patients who should receive systemic anticancer therapy, such as immunotherapy. There are different prognostic and predictive biomarkers, including tumor mutation burden, neoantigens, mismatch repair deficiency, microsatellite instability, TILs, and PD-L1 expression, which have been investigated by researchers in order to achieve the maximal efficiency of immunotherapy [124].

### 5.1 Genomic Biomarkers for Immunotherapy Response

#### 5.1.1 Tumor Mutational Burden

TMB has the potential to serve as a biomarker of response to immunotherapy in solid tumors, particularly ICIs. TMB, or tumor mutational burden, refers to the total number of mutations per megabase of tumor DNA and is increasingly recognized as a useful marker for guiding immunotherapy decisions. TMB is significant because it affects neoantigen production, proteins generated by variations in cancer-specific mutations. Neoantigens produced by these mutations can trigger an immune response. Tumors with high TMB generate more neoantigens compared to those with low TMB, making these neoantigens easier for the immune system to recognize and attack with the help of ICIs. The author highlights the correlation between high TMB and improved outcomes from ICIs. High-mutation cancers such as NSCLC, melanoma, and bladder cancer have benefited from immunotherapy. Cancers with higher mutation rates are more likely to produce neoantigens, increasing the chances of an immune response. The study emphasizes TMB’s potential to assist oncologists in identifying patients who may benefit from ICIs. Recently, pembrolizumab, a PD-1 inhibitor, was approved by the U.S. FDA for the treatment of solid tumors with high TMB regardless of the tumor's origin, reflecting a shift towards personalized cancer therapy based on tumor characteristics [125].

TMB is typically evaluated by targeted sequencing of tumors or blood. In addition to tissue-based TMB (tTMB), blood-based TMB (bTMB), which is non-invasive, is also gaining popularity. However, as previously noted, more work is required to standardize bTMB measurement and establish clear clinical decision cut-offs. This also highlights several challenges associated with the TMB application. One issue is tumor heterogeneity, in which different subclones within a tumor or metastatic lesions may have varying mutational loads. Another challenge is tissue heterogeneity, as different samples from the same tumor can yield different TMB values. Furthermore, TMB alone may not reliably predict therapy responsiveness, as tumors with low TMB might still be unresponsive to immunotherapy, even in the presence of PD-L1 expression or certain TME characteristics. Another limitation is the lack of universal TMB cut-off values across all cancer types; what is considered high TMB in one type of cancer may not be the same in another [126].

In conclusion, while TMB holds potential as a biomarker for immunotherapy, further research is necessary to refine its measurement and applicability across different tumor types. When used alongside other biomarkers and clinical factors, TMB can enhance treatment prognostication and support the development of precision cancer medicine.

#### 5.1.2 Microsatellite Instability

MSI has significant implications for cancer diagnosis, treatment, and outcome prediction, especially in metastatic colorectal cancer. MSI results from mutations in the DNA Mismatch Repair (MMR) system, which typically corrects replication errors in microsatellite regions—small, repetitive DNA sequences. The failure of this system leads to high mutation rates, particularly in genes that regulate cancer cell growth [127].

MSI detection is particularly important in colorectal cancer, where it serves as a biomarker for Lynch syndrome, an inherited condition that increases the risk of various cancers. MSI can be identified by PCR-based assays or immunohistochemical staining that detect loss of MMR proteins. For example, patients with MSI-H (high MSI) tumors in colorectal cancer typically exhibit better clinicopathological outcomes compared to those with MSS (microsatellite stable) tumors. MSI-H patients often experience fewer metastases and better overall survival rates [128].

In addition to its prognostic role, MSI has significant predictive value for immunotherapy. In this review, tumors exhibiting high microsatellite instability (MSI-H) in colorectal, gastric, and endometrial cancers respond exceptionally well to ICIs, particularly PD-1 inhibitors. This strong response is attributed to the high mutational burden of MSI-H tumors, which generates neoantigens, making the tumors more recognizable to the immune system and thus enhancing the efficacy of immunotherapy. As a result, MSI testing has become a critical component in evaluating patient eligibility for immunotherapy, with treatment decisions increasingly based on MSI status [129, 130].

#### 5.1.3 PDL-1 Expression

PD-L1, which is commonly referred to as Programmed Death Ligand-1, is one of the most important biomarkers in cancer treatment. It is a cell surface receptor present on tumors and specific immune cells. Once engaged with the PD-1 receptor on T cells, PD-L1 inhibits the immune system from attacking the tumor, thereby playing a very important role in cancer immune escape. Tumors expressing a higher level of PD-L1 are associated with better outcomes for immunotherapy drugs such as anti-PD-1 agents. These treatments counteract the signaling between PD-L1 and PD-1, thus allowing T cells to detect the tumor and launch an attack on it. PD-L1 expression depends on factors such as tumor type, the presence or absence of mutations, and the tumor microenvironment. However, there is ongoing debate regarding how the expression of this protein is regulated and altered after immune stimulation or inhibition and how it mediates immune suppression. Since PD-L1 can be induced by cytokines such as IFN-γ, PD-L1 levels may need to be monitored during treatment as they can fluctuate. PD-L1 expression may also vary within a tumor and be more prominent in some areas than in others, which reduces the effectiveness of PD-L1 as a single biomarker [131].

Additionally, PD-L1 expression varies within a tumor and may be more prominent in some areas than others, reducing PD-L1's effectiveness as a single biomarker. Therefore, it is recommended to use PD-L1 in combination with other biomarkers, such as TMB or MSI, to improve treatment accuracy. PD-L1 remains a promising biomarker; however, its effectiveness must be improved through the development of more sensitive testing platforms. Additionally, to enhance targeted cancer therapy, PD-L1 testing should be combined with other biomarkers [132].

#### 5.1.4 Neoantigen Landscape & Clonality

The neoantigen landscape comprises all variants of neoantigens arising from mutations in tumor somatic cells. All cancer cells contain clonal neoantigens, which emerge from driver mutations and direct immune resistance to tumor cells [133]. Immune checkpoint inhibitor sensitivity increases when neoantigens are detected in the patient. Greater percentages of clonal neoantigens within tumors lead to enhanced therapeutic responsiveness through PD-1/PD-L1 blockade because they generate T cell-mediated immune responses.

Research findings show that therapy response generally improves when patients present higher levels of neoantigens. The unique presentation of neoantigens elicits strong T-cell responses against tumors because these peptides are not expressed in normal tissues. The diversity of the T cell receptor (TCR) repertoire depends on neoantigen clonality levels because more diverse TCR landscapes are associated with improved therapeutic results. Immunoediting arises through immunogenic neoantigens, which activate adaptive immunity and create intra-tumor selective pressures that mold genetic profiles [134]. The process of immunoediting leads to the development of less immunogenic variants through immune recognition failure, thereby complicating treatment response.

#### 5.1.5 Tumor Immune Escape Mutations

These tumor immune escape mutations are genetic alterations that allow cancer cells to evade immune recognition and clearance. These mutations can affect the major histocompatibility complex (MHC) molecules and result in defective antigen presentation. Loss of heterozygosity (LOH) in *HLA* genes is a frequent mechanism of immune evasion, rendering tumors less detectable by cytotoxic T lymphocytes [135].

Furthermore, mutations in critical signaling pathways involved in immune recognition, including the interferon-gamma (IFN-γ) pathway, can diminish the antitumor efficacy of immune checkpoint inhibitors. Then, tumors with defects in the IFN-γ signaling pathway may have lower surface expression of PD-L1 and MHC molecules, further diminishing the immune response. Research into these immune escape mutations is essential in understanding resistance mechanisms and developing targeted therapies [135] ounteract immuno-hall pass [136].

In addition, specific mutations in tumor antigens that induce partial tolerance of T-cells, such as cancer/testis antigens, have been associated with resistance to checkpoint inhibitors, including PD-1 and CTLA-4 inhibitors. Detecting such mutations can inform how to tailor immunotherapy approaches to better benefit patients whose tumors employ immune evasion strategies [137].

#### 5.1.6 Epigenetic Biomarkers

Another emerging frontier in this regard is the use of epigenetic biomarkers to predict whether tumors respond to immunotherapy. These biomarkers result from heritable epigenetic changes in gene expression without nuclear mutations in their DNA [138]. DNA methylation, histone modifications, and other epigenetic modifications are important regulators of genes that can significantly impact the TME and immune landscape.

These changes in tumor epigenetic factors have also been associated with dysregulation of components in the TME that impact tumor immunogenicity, including tumor cells themselves and infiltrating immune cells. For example, changes in the expression of genes that mediate antigen presentation can also reduce T-cell recognition of tumor antigens. Epigenetic modulators have also shown efficacy in priming the antitumor immune response by enhancing antigen presentation. Proteins like BET can inhibit neoantigen presentation on the tumor cell surface, and this inhibitory activity can be reversed to restore T cell activity against tumorigenic cells [139].

This suggests that epigenetic therapy has the potential to sensitize tumors to immunotherapy, and recent studies indicate that immune checkpoint blockade, together with epigenetic therapies, may induce immunogenic cell death. It uses its ability to navigate the immunosuppressive tumor microenvironment, thereby enhancing overall response. This makes it possible to incorporate epigenetic biomarkers into clinical practice to improve patient stratification and achieve better outcomes in immunotherapy [140].

### 5.2 Clinical Trials and Genomic Profiling

Numerous clinical trials have been conducted to assess the role of genomics in predicting responses to immunotherapy. Data from these trials reveal significant associations between genomic markers and clinical outcomes, thereby validating the predictive power of genomic profiling in the context of cancer treatment, as shown in Table (**4**).

Beyond current biomarkers, emerging technologies continue to expand our ability to decode tumor–immune interactions.

### 5.3 Emerging Genomic Technologies in Immunotherapy

The era of high-throughput genomic technologies has brought about a revolution in cancer immunotherapy. These novel methods allow a better understanding of tumor biology among both researchers and physicians, which can ultimately be used to improve patient therapies.

#### 5.3.1 Next-generation Sequencing

High-throughput sequencing, particularly NGS (next-generation sequencing), is pivotal in advancing the understanding of cancer immunotherapy at the genomic level. NGS enables comprehensive analysis of tumor DNA, RNA, and immune interactions in a single run, making it easier to identify genetic patterns that predict a patient's response to immunotherapy. Notable features include the use of NGS to evaluate TMB and MSI, both established markers for predicting the effectiveness of immune checkpoint inhibitors [145].

Some studies have suggested that evaluating TMB or MSI-high scores enables oncologists to better select patients who may benefit from immunotherapy. NGS also provides insights into how new neoantigens—emerging antigens recognizable by the immune system—are identified, as well as other modifications in the TME that influence immunotherapy response. Furthermore, immunotherapy resistance profiles can be revealed by NGS. For example, mutations detected by NGS are useful for treatment prognosis, *e.g*., oncogenic alterations, and allow the determination of how tumor cells escape immune recognition; the mechanisms identified include defects in antigen presentation pathways [146].

This highlights the role of NGS in transforming precision medicine by enabling the tailoring of immunotherapy to tumor genomic characteristics. This approach significantly enhances the optimization of cancer therapies, ultimately leading to better clinical outcomes.

#### 5.3.2 Single-cell RNA Sequencing

Single-cell RNA sequencing (scRNA-seq) is an important method in immunotherapy that enhances the accuracy of treatment planning. Using scRNA-seq, researchers can uncover modifications within the TME and immune responses at the cellular level, as well as identify novel cellular subtypes that may be missed by traditional approaches. Additionally, the T Cell Receptor (TCR), particularly the CDR3 region, has been associated with T cell differentiation patterns and treatment response outcomes. scRNA-seq is crucial for identifying transcriptional regulation of immune checkpoint and multidrug resistance genes in response to immunomodulatory agents or tumor cells treated with chemotherapy at the single-cell level [147].

Additionally, scRNA-seq is valuable for identifying the transcriptional regulation of immune checkpoint and multidrug resistance genes following exposure to immunomodulatory agents or chemotherapy at the single-cell level [148]. This makes it an important tool in precision medicine, used to determine how various drugs affect specific immune cell populations. Additionally, scRNA-seq enables the identification of disease-associated shifts in cell composition and cellular state, thus offering a framework for therapeutic discovery directed toward novel cellular and molecular targets. It has been combined with CRISPR for screens, gene function analysis, and target identification. The ability of scalable gene knockdowns or edits provides a more comprehensive perspective of genetic networks and previously unrecognized cellular phenotypes, thus promoting cancer immunotherapy studies [149].

As an example, Kim *et al.* used scRNA-seq on CD45+ TILs from colorectal cancer mouse models and showed that Nt5e (CD73) and Entpd1 (CD39) influence TCR diversity and T-cell activation. They also demonstrated that TCR heterogeneity and Treg and macrophage populations were improved by PD-1 blockade and that AB680 improved the anti-cancer efficacy of immunosuppressed cells [150]. Similarly, Yu *et al.* [151] used scRNA-seq to profile mouse bone marrow progenitors and identified a subtype of innate lymphoid cell (ILC) precursors, PD-1hiIL-25Rhi progenitors, representing an initial checkpoint in ILC2 differentiation. They further demonstrated that PD-1 blockade perturbed ILC homeostasis and resulted in a decreased inflammatory response *in vivo*. These analyses emphasize how scRNA-seq can be utilized to identify new immune cell subsets, functions, and therapeutic targets, reinforcing the rationale for incorporating single-cell technologies into immunotherapy research.

#### 5.3.3 Multi-omics Approaches

Multi-omics approaches are able to merge different types of biological data (*e.g*., genomics, transcriptomics, proteomics, and metabolomics) to derive a more expansive and multi-dimensional understanding of tumor biology and immune-response mechanisms in the context of immunotherapies. Researchers can then integrate these various layers of data to uncover how these biological systems interact with one another and contribute to cancer progression and treatment efficacy [152].

One relevant innovation in this regard is spatial transcriptomics, which allows for the simultaneous analysis of gene expression and spatial localization of cells in tumor tissues [153]. This technique enables researchers to produce detailed maps of the TME, revealing heterogeneity that could affect treatment responses. These technologies provide an opportunity to study immune cell distribution relative to tumor cells and to understand immune evasion mechanisms of tumors. Identifying areas of immune cell infiltration compared with immune-excluded regions using spatial transcriptomics can reveal targets for therapeutic intervention and may enhance the effectiveness of immunotherapy approaches [154].

Moreover, the integration of multi-omics data also facilitates the identification of new biomarkers for predicting immunotherapy responses. For example, genomic alterations can be successfully integrated with transcriptomic profiles to predict the efficacy of ICIs in patients. By assessing these molecular profiles in conjunction with each other, we not only gain insights into the molecular basis of particular tumor types but also help guide the development of tailored therapies that can increase patient response.

#### 5.3.4 AI & Machine Learning in Immunogenomics

The emergence of artificial intelligence and machine learning algorithms in immunogenomics has grown exponentially, offering sensitive algorithms capable of analyzing large amounts of data generated by Next-Generation Sequencing (NGS). These algorithms leverage the capacity of deep networks to identify complex underlying patterns in genomic data, associating them with patient responses to ICIs, thereby allowing accurate predictions of treatment efficacy [155, 156].

A key area where AI is applied in this regard is to train models to distinguish between responders and non-responders to ICIs. AI-driven models can analyze genetic, transcriptomic, and clinical data to identify candidate patient-specific immunogenic mutations that may drive strong immune responses. These capabilities improve the specificity of immunotherapy by aiding clinicians in selecting candidates who are more likely to respond favorably to specific treatment protocols [157, 158].

Furthermore, AI may also help disentangle the interactions between immune cells and tumor cells in the TME. By leveraging large-scale data generated from multiple omics layers, AI algorithms can reconstruct complex biological interactions that can inform therapeutic responses, thus supporting the development of personalized approaches for targeting such interactions to tailor therapeutic strategies according to the biological composition of patients' tumors [159].

#### 5.3.5 Liquid Biopsy & Circulating Tumor DNA (ctDNA)

Liquid biopsy is a novel, non-invasive cancer diagnostic technique that utilizes ctDNA from the blood for analysis of genetic alterations in tumors, eliminating the need for tissue biopsies [160]. This strategy has several advantages, such as decreasing patient pain, lowering the risk involved, and allowing for real-time tracking of tumor progression and response to treatment. In fact, among lung cancer patients receiving ICIs, such as the PD-1 inhibitor pembrolizumab in particular, a high mutational discordance (based on ctDNA) has been associated with better outcomes. Moreover, fluctuations in ctDNA during treatment can indicate disease progression or resistance, prompting treatment adjustments [161].

In addition to their diagnostic applications, liquid biopsies can track dynamic tumor heterogeneity and clonal evolution over time, providing insights into emergent resistance mutations. Molecular variables are constantly changing, allowing for tailored treatment approaches. Using its ability to detect particular mutations, liquid biopsy allows for targeted therapy to be selected, ensuring optimal outcomes for the patient and also ensuring progress in the science of precision oncology [162]. Considering research and its utilization, these breakthroughs can be promising for the future of cancer management and treatment.

However, translating genomic discoveries into clinical success faces several challenges.

## 6 CHALLENGES AND LIMITATIONS

Tumor genomics is associated with numerous inherent challenges, including tumor heterogeneity, clonal evolution, and the complex surrounding liquid or tissue microenvironment; these factors can result in artificial representations of tumor dynamics and mechanisms [163]. Additionally, the efficacy of pharmacogenomics-based individualized cancer treatment is hampered by the genetic and phenotypic variability of tumor cells [164]. Genomics has revolutionized medicine, particularly in cancer treatment, by defining cancer by genetic alterations rather than tissue type. Cancer sequencing efforts, such as The Cancer Genome Atlas^7^ and the International Cancer Genome Consortium, have expanded our understanding of cancer mechanisms, molecular subtypes, and cancer heterogeneity. These data are easily accessible, allowing for clinical translation in areas such as recurrent mantle cell lymphoma and in categorized cancer subtypes for diagnosis, prognosis, and therapeutics [165].

### 6.1 Tumor Heterogeneity

Tumor heterogeneity now encompasses a wide range of factors that are associated with genetics. These factors pose a challenge to our understanding of cancer genesis. Malignant tumors are important aspects of their heterogeneity. Tumor heterogeneity can be classified as either temporal (changes in the molecular nature of cancer cells over time) or spatial (distribution of other gene groups within tumor cell subsets). This heterogeneity severely affects patient outcomes and the efficacy of anticancer therapies [166]. Tumor heterogeneity is influenced by both internal cell factors and the cell microenvironment.

Genomic instability is a major contributor to all malignancies, among many other variables that cause tumor heterogeneity, resulting in wide-ranging, random mutations in the genome. There are some examples that show the development of genomic instability. 1) The tumor suppressor p53 responds to DNA damage in normal cells, initiating pathways that lead to apoptosis, cell-cycle arrest, or DNA repair. These pathways are interconnected with tumor formation as well as genomic instability [92]. 2) In the tumor microenvironment (TME), hypoxic conditions are frequently observed. This is because higher oxygen demand is necessary for improved cellular kinetics and increased replicative potential [167]. Ma and colleagues collected the single-cell transcriptome landscape of 19 patients in one study, and liver cancer tumors with hypoxia-dependent VEGF expression showed greater transcriptome diversity. T cells with lower cytotoxic activity and worse overall survival were observed in tumors with greater heterogeneity. Hypoxia induces genomic instability, hinders mismatch repair, and promotes the development of subclones in the TME [168].

### 6.2 Resistance to Immunotherapy

The aim of all cancer treatments is to overcome cancer and achieve full recovery. Resistance to immunotherapy results from a decrease in the anticancer response following immunotherapy due to a number of intrinsic and extrinsic factors [169]. Resistance to immunotherapy occurs due to the following reasons.

#### 6.2.1 Antigen Presentation and Immunogenicity

Tumor cells have the ability to alter the amount of antigen or even completely lose it through a process known as antigenic drift, or they may simply change antigenicity so that they are not targeted by T cells. High TMB, MSI, and MMRd enhance neoantigen generation and T cell infiltration, but there is the possibility of immune evasion through endocytic antigen loss or antigen shedding. ER stress, as well as autophagy, mediate the immunogenic level of cellular death and result in resistance to immunotherapy [170-174].

#### 6.2.2 Antigen Presentation Pathway Disruptions

Genetic variants in proteasomes, transporters, and MHC molecules compromise antigen presentation, which in turn inhibits T cell activation and consequently promotes immune evasion. Epigenetic modifications may lead to suppression of antigen presentation and MHC gene expression [175-178].

#### 6.2.3 IFN-γ Signalling Pathway Alterations

Tumor cells may lose IFN-γ pathway function due to genetic or epigenetic changes. This includes mutations in JAK1/2 and STAT1, which are typically involved in phosphorylation events within transformed clones. Loss-of-function mutations in genes involved in the IFN-γ pathway lead to resistance to therapies such as PD-1 and CTLA-4 inhibitors. Other genetic alterations that inhibit IFN-γ signalling, including PTPN2, genes comprising the PBAF complex, and ADAR1, contribute to resistance by influencing immune responses and T-cell migration [179-181].

### 6.3 Limitations of Biomarkers

Biomarkers are biological indicators used to identify patients who should receive systemic anticancer therapy, such as immunotherapy. However, some biomarkers have limitations; for example, Tumor Mutational Burden currently lacks a standardized assessment technique and may differ across tumor types and locations. Microsatellite instability is a predictive biomarker restricted to specific tumor types. PDL1 expression shows limited sensitivity, specificity, and variability across tumor types and locations, and it changes over the course of treatment. TIL_s_ a prognostic biomarker. It has limited standardization in the quantification of TILs [182].

## 7 ETHICAL, LEGAL, AND SOCIAL IMPLICATIONS (ELSI) OF GENOMICS IN IMMUNOTHERAPY

Genomics in immunotherapy raises significant Ethical, Legal, and Social Implications (ELSI) that require careful consideration​.

Immunotherapy-related genomics raises ethical challenges around equity and access, as advanced therapies are unaffordable and available only in high-resource settings. Informed consent is also difficult due to the complexity of genomic information, while the sensitivity of genetic information poses a challenge to privacy and confidentiality. Sequencing finds incidental information that brings into question disclosure, and most treatments are experimental, raising a question of safety with innovation. In addition, the potential impact of genetic information on family members, along with the risk of generating false hope or unrealistic expectations of the new therapies, also complicates the ethical situation.

The legal implications of genomics of immunotherapy involve data protection, with regulations such as GDPR and HIPAA in place, but not universal worldwide. Intellectual property rights on biomarkers and targets might increase costs and restrict availability, and regulatory controls by agencies like the FDA, EMA, and CDSCO are crucial to ensure safety and validity. There is a risk of genetic discrimination by insurance companies or employers, and this is where stronger international legal protections are needed. Social implications of genomics in immunotherapy include ensuring that data remain secure in the eyes of a large part of the population, that minorities who feel their inclusion in the research is inadequate are not underrepresented, and that cultural and religious issues surrounding genetic testing are respected. Genomic outcomes may affect families and, in some cases, lead to stigma, stress, and difficult disclosure decisions. To address these issues, there needs to be greater education and awareness so that patients and society can make informed decisions about the use of precision immunotherapies. In addition, the incorporation of genomics into cancer care could affect the social acceptance of novel medical technologies, raise questions of fairness in the use of treatments that impose high financial costs, and inform communities' conceptions of the meaning of genetics in health and illness. Ensuring inclusivity and fairness in research participation and healthcare delivery is therefore vital to avoid widening existing social inequalities [183].

These challenges highlight the need for innovative solutions, which are addressed by emerging technologies and future directions in immunotherapy.

## 8 FUTURE PERSPECTIVE

The future of cancer immunotherapy lies in combining innovative strategies that enhance the body's natural ability to fight tumors. CRISPRCas9 technology, personalized neoantigen vaccines, and microbiome modulation are at the forefront of this revolution, offering new avenues for targeted and effective cancer treatments. This essay explores these strategies, focusing on their mechanisms and potential to transform cancer therapy.

### 8.1 CRISPR and Gene Editing

Immunotherapy is the most hopeful strategy among the current treatment modalities for cancer [184]. Techniques for generating chimeric antigen receptor T (CAR-T) cells were enhanced with the help of genome editing CRISPR^4^/Cas9. The technique relies on the fact that the patient's T-cells are taken and altered *ex vivo* so they can target cancer antigens during future infusions. Disruption of genes involved in T-cell signalling or inhibitory receptors by CRISPR/Cas9 enhances the function of CAR-T cell antagonists [185, 186]. The ability to use CRISPR/Cas9 in cellular transplantation to overcome differences in MHCs and replace big MHCs in their original locations is another advanced advantage of this technology [187]. For a Study Human primary CD4 + T cells were employed in a study to use CRISPR/Cas9 to knockout the B2M^5^ gene, which rendered cells devoid of MHC-I surface expression. Compared with the antigen genotypes of human leukocytes, this method is useful for generating transferable T cells that could benefit a range of cancer patients. In B-cell malignancies, CAR19 T cells have demonstrated strong biological activity against leukemic cells [188].

CRISPR/Cas9-mediated genome editing that removes genes encoding inhibitory T-cell receptors, such as CTLA-4 and PD-1. It could improve cancer immunotherapy with engineered tumor-reactive effector cells. This new technology is being tested for potential applications in cancer therapy. but is still in the experimental stage [189, 190]. Advancements in CRISPR/Cas9 technology have increased its potential benefits, making it a key therapeutic tool for various types of cancer. Clinical trials are being conducted on CRISPR/Cas9 technology to assess its efficacy and safety [191]^6^.

CRISPR-Cas9 technology is utilized to reshape anti-tumor immunity by boosting tumor antigenicity. It may include increasing tumor immunogenicity and enhancing host immunity [192]. For example, CRISPR knockout of the immune checkpoint molecule PD-L1 in glioblastoma cells has been shown to significantly enhance killing by T cells [193]. CRISPR/Cas9 dual-sgRNA knockout of PD-L1 in human U87 glioblastoma tumor cells inhibits cell proliferation, cell invasion, and tumor-associated macrophage polarization [193]. This precise editing enables the immune system to more effectively target tumor cells. In addition, CRISPR-based T-cell engineering has led to the development of CAR-T and TCR-T cell therapies; these treatments enhance specific immune responses, minimize off-target effects, and improve efficacy even in solid tumors. CRISPR/Cas9 enhances the specificity and efficiency of Chimeric Antigen Receptors (CARs) and T-Cell Receptors (TCRs) targeting tumor-associated antigens, thereby boosting the immune system’s ability to kill cancer cells [194].

### 8.2 Combination Therapy

Preclinical and clinical research have demonstrated encouraging outcomes when various combinations of immunotherapy agents are used, as well as when immunotherapy is combined with conventional therapies such as radiotherapy or chemotherapy. For example, individuals with melanoma and renal cell carcinoma show improved response rates when anti-PD-1/PD-L1 medicines are used with anti-CTLA-4 drugs. Similarly, patients with BRAF-mutant melanoma have shown better outcomes when immunotherapy is combined with targeted therapies such as BRAF inhibitors. Furthermore, by releasing tumor antigens and promoting immune cell infiltration, combining immunotherapy with local therapies such as radiation therapy can enhance the immune response. Consequently, combination therapies are expected to remain a major focus of immunotherapy research in the future [195].

Combined therapies, like anti-CTLA-4 and other anti-PD-1 antibodies, show remarkable success and patients' survival rate in various forms of cancer. These T-cell immune checkpoint molecules, which control antitumor immunity, have transformed cancer therapy. Scientific research has shown that both CTLA-4 and PD-1 checkpoint inhibitors improve patient survival in diverse relapsed cancers, including melanoma, renal cell carcinoma, squamous cell carcinoma, NSCLC, and others. Relative to other chemotherapeutic modalities, hence increasing its efficacy in the treatment success and survival rates [196].

Other therapy such as stereotactic body radiation therapy (SBRT): It has been reported that combined SBRT with PD-1 inhibitors has shown appreciable effectiveness in eradicating tumors among patients with nasopharyngeal carcinoma. This technique is an advanced radiation technique in which radiation is delivered accurately to cancer cells over a short period (ranging from 1 to 5 fractions, typically over 1 to 2 weeks). It also delivers a high dose of radiation, often above 5 Gy per fraction. Many clinical trials have enhanced treatment effectiveness and reduced toxicity with the use of chemoradiotherapy combined with immunotherapy, which has had a profound impact on tumor metastasis [197].

### 8.3 Personalized Medicine

A potential therapeutic approach involves combining neo-epitope-based vaccines with oncolytic virotherapy. For each patient, neo-epitopes are utilized to develop personalized vaccines that effectively stimulate CD8+ T cell recognition of tumor cells [198]. The current study involves targeting and destroying cancer cells while modifying the TME by employing oncolytic vaccinia viruses (VV). This also involves releasing cytokines and immune checkpoint inhibitors to increase T cell infiltration and activity. Following this, personalized vaccines combined with oncolytic viruses aim to overcome the immunosuppressive TME by enhancing the sensitivity of the immune system in targeting and eliminating cancer cells. This approach involves identifying new antigens through advanced genomics, transcriptomics, and proteomic profiling, with the aim of tailoring treatments for individual patients to avoid relapse and effectively manage metastatic triple-negative breast cancer (TNBC) [199].

#### 8.3.1 Personalized Neoantigen Vaccines

Personalized neoantigen vaccines represent a breakthrough in cancer immunotherapy, and clinical trials of mRNA-based vaccines targeting tumor-specific mutations have reported promising outcomes. These vaccines contain genetic information encoding tumor-specific antigens and are processed and translated into proteins by the patient’s own cells, leading to an immune response [200]. While both approaches have produced positive results, combinations of the two have shown improved efficacy, as demonstrated by Moderna’s mRNA-4157, which showed a positive impact in patients with melanoma when paired with immune checkpoint inhibitors. mRNA-4157/V940 plus KEYTRUDA as adjuvant therapy reduced the risk of recurrence or death by 44% compared with KEYTRUDA alone [201]. These vaccines are designed to prepare the immune system to produce a specialized antitumor response based on the mutation signature of a patient’s tumor. Neoantigen selection is increasingly complemented by computational algorithms to maximize vaccine potency and specificity [202].

The algorithms help scientists identify specific immunogenic neoepitopes, which leads to enhanced performance of personalized cancer vaccines.

### 8.4 Microbiome-Genomic Interactions

Scientists now understand how the gut microbiome affects immunotherapy responses, which provides opportunities to enhance therapeutic outcomes through microbiome modification. Data show that treatment with PD-1 inhibitors works better for patients who have Faecalibacterium-rich communities [203]. High levels of the Faecalibacterium genus were identified as the most significant indicator for positive ICI responses in a meta-analysis. Researchers predict that upcoming cancer treatments will combine microbiome modification techniques with genetic-based immunological therapies for improved disease management [204]. The combination of ICIs with FMT in clinical trials positively affects treatment success rates [205]. The sustained impact of FMT on patient microbiota triggered beneficial clinical changes in 6 out of 13 monitored patients, including 1 partial response and 5 cases of stable disease, resulting in objective response and disease control rates of 7.7% and 46.2%. The clinical response observed in this study correlated with higher levels of immune cytokines and cytotoxic T cells in both peripheral blood and tumor tissues [206].

## CONCLUSION

In conclusion, genomics has revolutionized cancer immunotherapy by deepening our understanding of tumor biology, patient-specific differences, and treatment response. Biomarkers like TMB and MSI, along with advanced technologies such as CRISPR/Cas9 and single-cell sequencing, have advanced precision therapies. Understanding the tumor microenvironment and its genomic alterations has revealed resistance mechanisms and novel therapeutic opportunities. In addition to these advances, however, difficulties like variability among patients, ethical concerns, and requirements for standardization persist. The future holds the prospect of combining multi-omic data to enhance the capture of tumor-immune complexity and of leveraging AI-based predictive models to guide treatment outcomes. These technologies promise to improve patient stratification, personalize therapies, and ultimately increase the overall efficacy of cancer immunotherapy.

## Figures and Tables

**Fig. (1):**
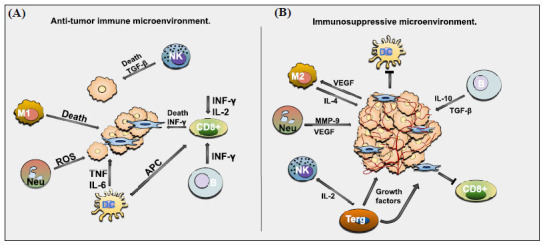
Comparison of anti-tumor *versus* immunosuppressive tumor microenvironments. (**A**) In the anti-tumor environment, M1 Macrophages (M1), neutrophils (Neu), Dendritic Cells (DC), Natural Killer Cells (NK), and cytotoxic T lymphocytes (CD8^+^) release Reactive Oxygen Species (ROS), TNF, IL-6, and IFN-γ, promoting tumor cell death. (**B**) In the immunosuppressive microenvironment, M2 macrophages (M2), regulatory T cells (Treg), neutrophils (Neu), and B cells release immunosuppressive cytokines (IL-4, IL-10, TGF-β) as well as VEGF, which stimulate angiogenesis and suppress CD8^+^ activity. Abbreviations: APC = antigen-presenting cell; VEGF = vascular endothelial growth factor; IL = interleukin; TGF-β = transforming growth factor-beta. Schematic representation created by the authors based on concepts from the literature.

**Fig. (2):**
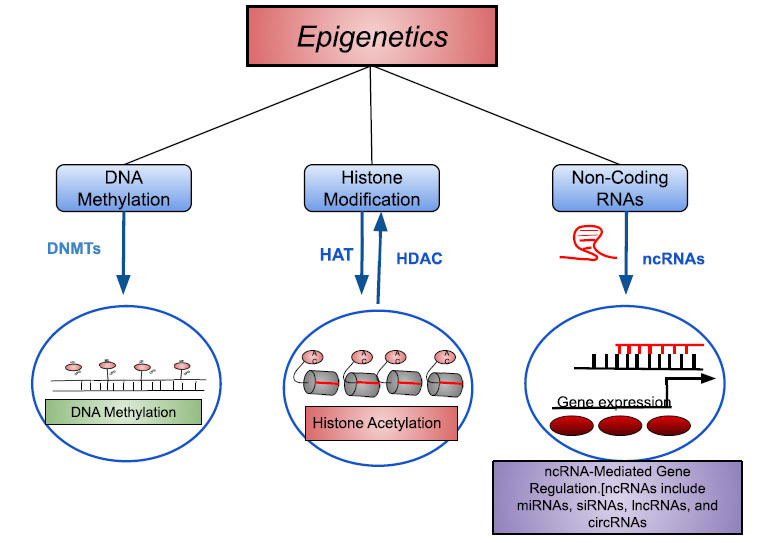
Major epigenetic modifications that regulate gene expression and their biological mechanisms. DNA methylation, histone acetylation/methylation, and chromatin remodeling are shown. Abbreviations: DNMT = DNA methyltransferase, HDAC = histone deacetylase. Schematic representation created by the authors based on concepts from the literature.

**Fig. (3):**
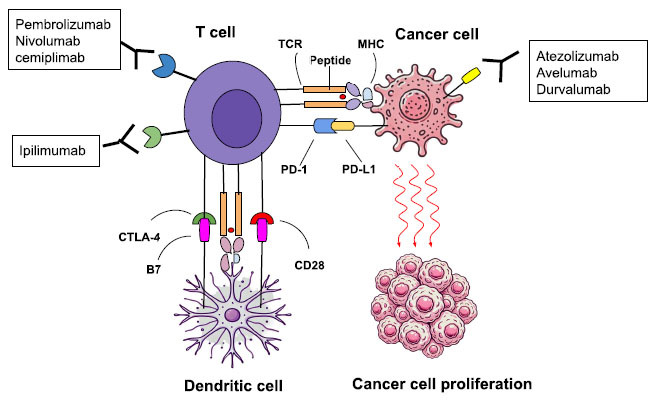
Immune checkpoint inhibitors approved by the FDA. Schematic representation created by the authors based on concepts from the literature.

**Fig. (4):**
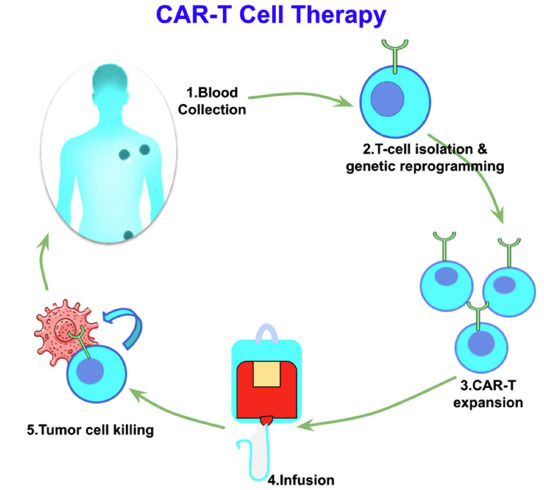
CAR T cell therapy. Schematic representation created by the authors based on concepts from the literature.

**Fig. (5):**
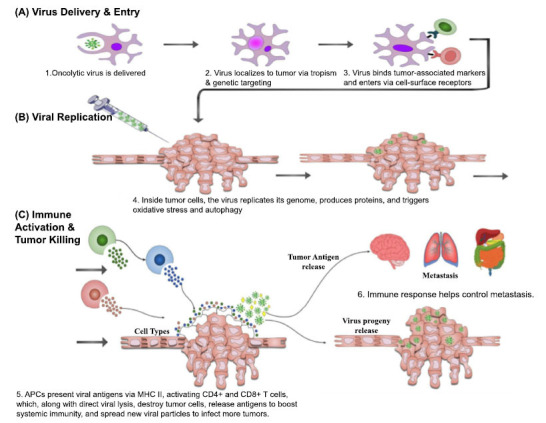
Mechanism of action of oncolytic virus. Schematic representation created by the authors based on concepts from the literature.

**Table 1:** FDA-approved immune checkpoint inhibitors that are currently undergoing clinical studies.

Targets	Drug	Class	Approval Year	Indication(s)	References
CTLA-4	Ipilimumab	IgG1 mAb	2011	Melanoma	[76]
PD-1	Pembrolizumab	IgG4 mAb	2014	Melanoma, NSCLC, HNSCC, MSI-H solid tumors, GC, cervical cancer, urothelial carcinoma, TNBC, TMB-H/dMMR endometrial carcinoma.	[77]
	Nivolumab	IgG4 mAb	2014	Melanoma, NSCLC, RCC, Hodgkin’s lymphoma, HNSCC, HCC, ESCC, pleural mesothelioma, MSI-H/dMMR CRC.	[78, 79]
	Cemiplimab	IgG4 mAb	2018	Cutaneous squamous cell carcinoma.	[80]
PDL-1	Atezolizumab	IgG1 mAb	2016	Urothelial carcinoma, NSCLC, SCLC, melanoma, HCC.	[81]
	Durvalumab	IgG1 mAb	2017	Urothelial bladder cancer, NSCLC, SCLC, BTC.	[82]
	Avelumab	IgG1 mAb	2017	Merkel cell carcinoma, urothelial carcinoma, RCC.	[83]

**Abbreviations:** CTLA-4 = Cytotoxic T-lymphocyte-associated protein 4; PD-1/PD-L1 = Programmed cell death protein-1 and its ligand-1, respectively; IgG = Immunoglobulin G; NSCLC = Non-small-cell lung cancer; HNSCC = Squamous cell carcinoma of the head and neck; GC = Gastric cancer; TNBC = Triple-negative breast cancer; RCC = Renal cell carcinoma; HCC = Hepatocellular carcinoma; SCLC = Small-cell lung cancer; ESCC = Esophageal squamous cell carcinoma; BTC = Biliary tract cancer; CRC = Colorectal cancer; TMB-H = Tumors with high mutational burden; dMMR = Mismatch repair deficient; MSI-H = high microsatellite instability.

**Table 2:** Treatment modalities for adoptive cell therapy.

-	TIL	TCR	CAR
First evidence of clinical benefit	1994	2006	2013
Production method	T cell isolation from cancers and *ex vivo* growth	Peripheral T cell isolation *via* apheresis and *ex vivo* TCR-based transduction against tumor antigen	Peripheral T cell isolation by apheresis and *ex vivo* CAR-mediated tumor antigen transduction
Target	It works on complexes like MHC-peptide	It works on complex MHC-peptide	It works on surface protein cells, which are non-MHC cells
Lymphodepleting preparative regimen	Yes	Yes	Yes
Specificity	It is specific to polyclonal	It is specific to Monoclonal	It is specific to Monoclonal
Main toxicity	Lymphodepleting regimen *IL-2* mediated (chills, fever, edema) Seldom autoimmune	Lymphodepleting regimen “On-target, off-tumor” CRS	Lymphodepleting regimen “On-target, off-tumor” CRS Neurological
Restrictions	Complex Heterogeneous infusion product	MHC-restricted Currently not yet tumor-specific Toxicity	Currently only effective for the treatment of haematological malignancies Toxicity

**Note:** * – TIL = Tumor-infiltrating lymphocyte; TCR = T-cell receptor; CAR = Chimeric antigen receptor; MHC = Major histocompatibility complex; *IL-2* = Interleukin-2; CRS = Cytokine release syndrome. ‘On-target, off-tumor’ is a situation where immune molecules target the healthy cells, which have similar antigens to the tumor-associated ones, and thus result in toxicity. Lymphodepleting regimens are the pretreatment regimens that are used to increase the effectiveness of adoptive cell therapies.

**Table 3:** Different types of therapeutic vaccines, along with their mechanisms of action for specific cancer types.

Vaccine Method	Targets	Mechanism	Vaccine Type	Type of Cancer
Adoptive T cell transfer	Tumor-associated antigen	The Response is based on CTL	Cell-based	Prostate cancer, metastatic melanomas
Allogenic whole cancer cells with BCG stimulant	All tumor cell antigens	It works by stimulating the T cells, NK cells, macrophages, and eosinophils.	Cell-based	Prostate cancer, heterogeneous metastatic melanoma
APC-based vaccine	Antigen associated with the tumor	The response is based on CTL activation of DCs, which should then mature.	Cell-based	Metastatic prostate cancer
DNA	Antigen associated with the tumor	The response is based on activation of CTL as well as Antibody	Antigen-based	Melanoma, mammary carcinoma, colon and lung carcinoma
Proteins	Carbohydrates associated with tumors	Antibody development, T cell response	Antigen-based	Melanoma, lung cancer
Whole bacteria	Bacterial antigen	The response is based on CTL as well as on Antibody by immune stimulation	Antigen-based	Leukemia, bladder cancer, lung cancer, melanoma, and other neoplasms

**Note:** * - CTL = Cytotoxic T lymphocyte; NK = Natural killer cells; APC = Antigen-presenting cell; DC = Dendritic cell; BCG = Bacillus Calmette–Guérin. We have terms such as “Cell-based,” which refers to vaccines that are derived from whole cells that are meant to stimulate an immune response, and “Antigen,” which refers to types of vaccines that use specific antigens to trigger an immune response.

**Table 4:** Clinical trials highlighting the integration of genomic profiling in cancer immunotherapy for improved patient stratification and treatment outcomes.

Trial Name/ID	Cancer Type	Genomic Biomarker	Immunotherapy Used	Phase	Findings/Outcome	References
**KEYNOTE-158**	NSCLC, MSI-H Tumors	MSI-H/dMMR	Pembrolizumab	Phase II	Improved response rate in MSI-H patients	[141]
**CheckMate 227**	NSCLC	TMB (≥10 mut/Mb)	Nivolumab + Ipilimumab	Phase III	Higher PFS in the high TMB group	[142]
**IMPower150**	NSCLC	*STK11* mutation	Atezolizumab + Chemo	Phase III	*STK11* mutations linked to poor response	[143]
**JAVELIN 100**	Urothelial Carcinoma	PD-L1 Expression	Avelumab	Phase III	OS benefit in the PD-L1+ subgroup	[144]

## Footnotes

1 *Oxidative stress- It is the condition where there is an imbalance between reactive oxygen species (ROS), and the antioxidants. It contributes to aging and such diseases as cancer and neurodegeneration.

2 The Human Genome Project (1990–2003) completely changed the field of genomics through the mapping of the human genome and enables cancer researchers to study gene alterations.

3 Epigenetic therapy remains a relatively novel approach in cancer therapy, involving basically modifiable processes such as DNA methylation and histone acetylation.

4 Clustered Regularly Interspaced Short Palindromic Repeats, is a revolutionary gene editing tool that uses the Cas9 protein to make a specific cut in DNA and allow gene modification.

5 CRISPR/Cas9-mediated knockout of the B2M gene results in the absence of MHC-I on the cell surface, and this can aid in the prevention of immune rejection in cellular transplantation.

6 So other than cancer, CRISPR/Cas9 has widespread applications, such as treating genetic disorders as well as autoimmune diseases.

## LIST OF ABBREVIATIONS

CAF: Cancer-Associated Fibroblast
CCL7: C-C Motif Chemokine Ligand 7
CTGF: Connective Tissue Growth Factor
CXCL2: C-X-C Motif Chemokine Ligand 2
ECM: Extracellular Matrix
EGFR: Epidermal Growth Factor Receptor
HGF: Hepatocyte Growth Factor
HIF: Hypoxia-Inducible Factor
HNSCC: Head and Neck Squamous Cell Carcinoma
ICI: Immune Checkpoint Inhibitors
IGF: Insulin-Like Growth Factor
IL-6: Interleukin-6
MDSC: Myeloid-Derived Suppressor Cell
MSI: Microsatellite Instability
NK: Natural Killer (cells)
NSCLC: Non-Small Cell Lung Cancer
PD-L1: Programmed Death-Ligand 1
TCR: T-Cell Receptor
TGF-β: Transforming Growth Factor Beta
TIL: Tumor-Infiltrating Lymphocyte
TMB: Tumor Mutational Burden
TME: Tumor Microenvironment
Tregs: Regulatory T cells
VEGF: Vascular endothelial growth factor

## GLOSSARY

1. Tumor microenvironment - The complex environment surrounding tumor cells, which consists of immune cells, stromal cells, and extracellular matrix, plays an important role in tumor progression and response to treatment.
2. Cancer immunotherapy- A treatment strategy that enables the immune system to recognize cancer cells and destroy them.
3. Tumor Mutational Burden: A measure of the number of mutations per megabase pair of DNA in a tumor and used as a biomarker to predict responses to immunotherapy.
4. Microsatellite instability - It is a condition of genetic hypermutability caused by defects in the mismatch repair system and used as a predictive marker for some types of cancer treatments.
5. Checkpoint inhibitors: These are the drugs that block proteins like PD-1 or CTLA-4 to reactivate T-cells against cancer cells.
6. Neoantigen: New antigens derived from mutations in tumor cells and recognized by the immune system.
7. Next-Generation Sequencing (NGS): A high-throughput DNA sequencing technology used to analyse the genetic and molecular profile of tumors.
8. Cancer-Associated Fibroblasts (CAFs): One of the cellular components of the tumor microenvironment that enhances the growth of the tumor and has the ability to influence treatment response.
9. Epigenetics: Study of changes in gene expression that do not involve changes in the DNA sequence and lead to cancer development.
10. Single-Cell RNA Sequencing (scRNA-seq): An approach to study the gene expression profile at the single-cell level and have application in TME heterogeneity and immune response analysis.
